# Exercise induction of gut microbiota modifications in obese, non-obese and hypertensive rats

**DOI:** 10.1186/1471-2164-15-511

**Published:** 2014-06-21

**Authors:** Bernardo A Petriz, Alinne P Castro, Jeeser A Almeida, Clarissa PC Gomes, Gabriel R Fernandes, Ricardo H Kruger, Rinaldo W Pereira, Octavio L Franco

**Affiliations:** Centro de Análises Proteômicas e Bioquímicas, Programa de Pós-Graduação em Ciências Genômicas e Biotecnologia da Universidade Católica de Brasília, Brasília, DF Brazil; Laboratorio de Enzimologia, Departamento de Biologia Celular, Universidade de Brasília, Brasília, DF Brazil; Programa de Pós-Graduação em Educação Física, Universidade Católica de Brasília, Brasília, DF Brazil; Pos-Graduação em Biotecnologia, Universidade Católica Dom Bosco, Campo Grande, MS Brazil; UDF centro Universitário, Brasília, DF Brasil

**Keywords:** Gut microbiota, Obesity, Hypertension, Exercise, Lactobacillus

## Abstract

**Background:**

Obesity is a multifactor disease associated with cardiovascular disorders such as hypertension. Recently, gut microbiota was linked to obesity pathogenesisand shown to influence the host metabolism. Moreover, several factors such as host-genotype and life-style have been shown to modulate gut microbiota composition. Exercise is a well-known agent used for the treatment of numerous pathologies, such as obesity and hypertension; it has recently been demonstrated to shape gut microbiota consortia. Since exercise-altered microbiota could possibly improve the treatment of diseases related to dysfunctional microbiota, this study aimed to examine the effect of controlled exercise training on gut microbial composition in Obese rats (n = 3), non-obese Wistar rats (n = 3) and Spontaneously Hypertensive rats (n = 3). Pyrosequencing of 16S rRNA genes from fecal samples collected before and after exercise training was used for this purpose.

**Results:**

Exercise altered the composition and diversity of gut bacteria at genus level in all rat lineages. Allobaculum (Hypertensive rats), Pseudomonas and Lactobacillus (Obese rats) were shown to be enriched after exercise, while Streptococcus (Wistar rats), Aggregatibacter and Sutturella (Hypertensive rats) were more enhanced before exercise. A significant correlation was seen in the Clostridiaceae and Bacteroidaceae families and Oscillospira and Ruminococcus genera with blood lactate accumulation. Moreover, Wistar and Hypertensive rats were shown to share a similar microbiota composition, as opposed to Obese rats. Finally, *Streptococcus alactolyticus, Bifidobacterium animalis, Ruminococcus gnavus, Aggregatibacter pneumotropica and Bifidobacterium pseudolongum* were enriched in Obese rats.

**Conclusions:**

These data indicate that non-obese and hypertensive rats harbor a different gut microbiota from obese rats and that exercise training alters gut microbiota from an obese and hypertensive genotype background.

**Electronic supplementary material:**

The online version of this article (doi:10.1186/1471-2164-15-511) contains supplementary material, which is available to authorized users.

## Background

Exercise practice is a non-pharmacological treatment for a series of diseases [1]. Along with dietary control, appropriate exercise programs are proposed to treat and attenuate obesity [2] and its associated cardiovascular disorders such as hypertension [3]. It is known that hypertension and obesity frequently coexist [4], affecting millions of people worldwide [5].

Gut microbiota have been recently indicated as having a close relationship with obesity, where the microbiota of an obese subject presents an enhanced ability to harvest energy and accumulate fat [6]. The gut harbors the greatest density of these microorganisms in the body (e.g. ~up to 1.5 kg in the human gut) [7] with Firmicutes, Bacteriodetes and Actinobacteria constituting the dominant phyla [8]. Moreover, obesity is associated with reduced microbiota diversity at phylum level [9], seen in rodents and in humans [10, 11].

The gut is a dynamic environment, highly exposed to environmental factors such as diet [12], antibiotics [13], pathogens [14] and lifestyle [15], which constantly interact with microbial communities. In addition, gut microbiota is also shaped throughout life by host-related factors such as host-genotype [16]. Disturbance within gut microbiota has been reported to influence host susceptibility to pathogens and pathological conditions such as gastrointestinal inflammatory diseases and obesity [17]. Moreover, hypertension induction was also seen to alter gut microbiota [18].

It has been proposed that dysbiosis and pathologies associated with unbalanced gut microbiota may be prevented or treated with prebiotics, probiotics and fecal microbiota transplantation [19, 20]. In addition, controlled exercise intensities are related to protective effects on the gastrointestinal tract, including a reduced risk of colon inflammation and cancer [21]. It is proposed that exercise may reduce intestinal transit time, diminishing the contact between the colon and cancer-promoting agents [22].

Recently, exercise has also been shown to induce alterations within microbiota composition [22, 23], which suggests that exercise may be included as a possible therapeutic factor along with diet, prebiotics and other treatments. Since exercise plays a prominent role in metabolic regulation and energy expenditure, it might modulate host-microbiota interaction, affecting the host metabolism. Although these relations are still unknown, exercise may enhance the strategies for obesity control, along with other actions such as microbiota transplant [20].

Although voluntary exercise was shown to alter microbiota in non-pathological animals [22–24], its effects on gut microbiota still need to be further investigated in pathologic phenotypes and through controlled parameters such as exercise volume and intensity. Therefore, in the present study, we proposed to examine the effect of controlled moderate exercise intensity on gut microbial status in rats with different phenotypes, by using pyrosequencing of 16S RNAs genes from fecal microbiota samples. To our knowledge, this is the first study to use controlled exercise parameters and distinct animal strains with different obesity and hypertension genotypes.

Analyzing 16S rRNA sequences revealed a similar microbiota profile shared between Wistar and Hypertensive rats, with both being divergent from Obese rats. Exercise was shown to enhance bacterial diversity and to alter microbial communities at the species level in all animal lineages. Thus, these data contribute to the emerging knowledge regarding the effect of exercise on gut microbiota, but further studies should be performed to establish the mechanism by which exercise signals in the bacterial community and to determine the impact of these modulations on host homeostasis.

## Methods

### Animals

Animals were obtained from the Federal University of São Paulo, Brazil (UNIFESP) and started the experiment at ~18 weeks of age. Three different strains from two different genotypes were used: an obese genotype, homozygous (fa/fa) obese Zucker rats [25] (Obese rats; n = 5, 389.4 ± 21 g) and a hypertensive genotype, spontaneous hypertensive rats (Hypertensive rats; n = 5, 227.4 ± 29.3 g), a strain obtained by the selective breeding of Wistar-Kyoto rats with high blood pressure [26]. A strain of Wistar rats (WR; n = 5, 223.2 ± 27.3 g) was used as normotensive control for SHR and as a non-obese phenotype [26] (Additional file 1). All animals were allocated to collective cages according to their lineages, being kept in a 12 h light–dark cycle environment, with food and water ad libitum*.* All experimental procedures and interventions in the present study, involving animal-welfare, were carried out in strict accordance with the recommendations in the Guide for the Care and Use of Laboratory Animals of the National Institutes of Health and were approved by the local ethics committee for standards in animal use, at the Institute of Biological Sciences, University of Brasilia, Brazil (UnBDOC no. 48695/2010); these were also in accordance with international standards. After experimental procedures, all animals were deeply anesthetized with 2% Xylazine (50 mg.kg^−1^) and 10% Ketamine (80 mg.kg^−1^) and euthanized by cervical dislocation. Through the entire experiment, all efforts were made to minimize animal suffering.

### Exercise training

Before the training period, all animals underwent a familiarization period on a treadmill device (Li 870, Letica Scientific Instruments, Barcelona, Spain) for 2 weeks to reduce external stress. Duration and speed on treadmill were increased progressively (up to 12.5 m.min^−1^ for Obese rats; 20 m.min^−1^ for Hypertensive and Wistar rats) as previously described [27, 28]. In addition, blood pressure of Wistar and Hypertensive rats was measured by the tail-cuff method [29] at the beginning of the experiment to characterize the hypertensive phenotype from the SHR strain (171.4 ± 7.7 mm.Hg^−1^ for Hypertensive rats and 128 ± 5.9 mmHg^−1^ for Wistar rats) (Additional file 2). As regards the Obese rats group (obese (fa/fa) Zucker rat), these animals are homozygous for the *fa* allele and are one of the most common models of genetic obesity where rats normally exhibit hyperlipidemia, hyperinsulinemia, hyperphagia and a significant weight gain by the 3rd to the 5th week of age [25]. In this model, conflicting results were reported on whether these obese Zucker rats are hypertensive compared to the lean control rats, showing that blood pressure is not elevated in this model [30, 31]. However, the obesity phenotype may enhance arterial peripheral resistance and the animal may develop hypertension secondary to obesity and to secondary mechanisms [32, 33]. Considering that obese Zucker rats do not present a regular pattern of blood pressure as reported by different studies, the systolic blood pressure was not measured in this group.

After the adaptation period, all animals were trained for 30 min per day, 5 days per week for 4 weeks. Running intensity was set corresponding to maximal lactate steady state (MLSS), previously identified in obese Zucker rats [28] and SHR rats [27]. Therefore, for Obese rats, running velocity was set at 12.5 m.min^−1^ and for Hypertensive and Wistar rats at 20 m.min^−1^. A new MLSS identification was performed after the fourth week of exercise training to assess each animal’s cardiovascular adaptation.

### Blood lactate analysis

Capillary blood samples (10 μL) were collected through a small incision in the distal portion of the tail of the animals at rest and every 5 min during the MLSS test. Capillary blood samples were placed in microtubes (0.6 mL) containing 20 μL of 1% sodium fluoride and stored at −20°C. Analyses were performed by electro-enzymatic method with YSI 1500 Sports (Yellow Springs, OH, USA). The MLSS was considered when there was no increase over 1 mmol.L^−1^ of blood lactate from 10 to 25 min of exercise tests [34].

### Fecal DNA extraction and barcoded pyrosequencing of the 16S rRNA gene

Fecal content from all animals was individually collected in three replicates before exercise training period and in vivo 24 h after the last session of exercise (Additional file 1). Fecal samples were stored in RNA later® (Life technologies) until samples were frozen at −80°C. Three out of five individual fecal samples from each animal lineage were randomly selected. Fecal microbial DNA was extracted from ~0.25 g using the PowerFecal DNA Isolation Kit (MoBio, Carlsbad, CA, USA) according to the manufacturer’s instructions. The triplicate DNA extractions were not pooled.

The bacterial community partial 16S rRNA gene was amplified with primer pair 787 F-1492R [35]. For pyrosequencing analysis, the primer set was modified, where the forward primer included the Roche 454-A pyrosequencing adapter and a 12-bp barcode (unique to each sample), while the reverse primer included only the Roche 454-B pyrosequencing adapter. The 20 μl reaction mixture for the PCR contained approximately 10 ng of metagenomic fecal DNA, 1X PCR buffer (Invitrogen), 3.0 mM MgCl2, 10 pmol of each primer, 0.25 mM dNTP, and 1.5 U Taq DNA polymerase (Invitrogen). The cycling protocol started with an initial denaturation step of 3 min at 95°C, followed by 25 cycles of denaturation for 30 s at 95°C, annealing for 30 s at 58°C, and extension for 1.40 min at 72°C, followed by a final extension for 7 min at 72°C and cooling to 10°C. Finally, the rRNA amplicons from bacterial communities were purified with the QIAquick PCR Purification Kit (Chatsworth, CA). The concentrations of the rRNA amplicons were measured by Qubit fluorometer (Invitrogen), and subsequently the massively parallel GS FLX Titanium technology was performed in the Roche 454 Life Sciences Corporation, Branford, CT, USA.

### Analysis of 16S rRNA sequences

A total of 1,398,681 16S rRNA sequences were obtained by the 454 GS FLX Titanium sequencer. All the 16S amplicons were processed by the quantitative insights into microbial ecology (QIIME) pipeline version 1.6.0-dev [36]. Briefly, all the 16S rRNA amplicons were sorted by their barcodes, and subsequently the reads with a length less than 180 bp, ambiguous sequences, bases with Phred values of <30 and their primers, barcodes and adaptor sequences were removed. The remaining sequences were submitted to Denoiser algorithm [37] to remove pyrosequencing errors. Operational taxonomic units (OTUs) were clustered at 97% similarity using an ‘open-reference’ OTU picking protocol, where sequences are clustered against the Greengenes database [38] using Uclust [39]. One of the most-abundant reads from each OTU was aligned using the PyNAST algorithm [36]. The chimeric OTUs were detected with ChimeraSlayer [40], and taxonomic classifications were assigned with the naïve Bayesian classifier of the Ribosomal Database Project (RDP) classifier [41] applying 80% of confidence threshold. Shannon indices and observed richness were used to evaluate community richness, and the unweighted Unifrac algorithm was performed to generate principal coordinate plots (PCoA).

### Statistical analysis

Statistical differences between the groups pre-exercise and after exercise were tested using the analysis of similarities (ANOSIM) by permutation of group membership with 999 replicates [42], and bivariate relationships were measured with Pearson correlations and regression analysis available through QIIME [36]. Statistical tests on the taxonomic differences between samples were calculated by Welch’s *t*-test combined with Welch’s inverted method for calculating confidence intervals (nominal coverage of 95%), using the Statistical Analysis of Metagenomic Profiles (STAMP) software version 2.0.0 (STAMP) [43].

### Accession number

The 454 FLX Titanium flowgrams (sff files) have been submitted to the National Center for Biotechnology Information (NCBI) Sequence Read Archive database, project number: PRJNA246617.

## Results

### Effect of exercise training on aerobic capacity

**Figure 1:**
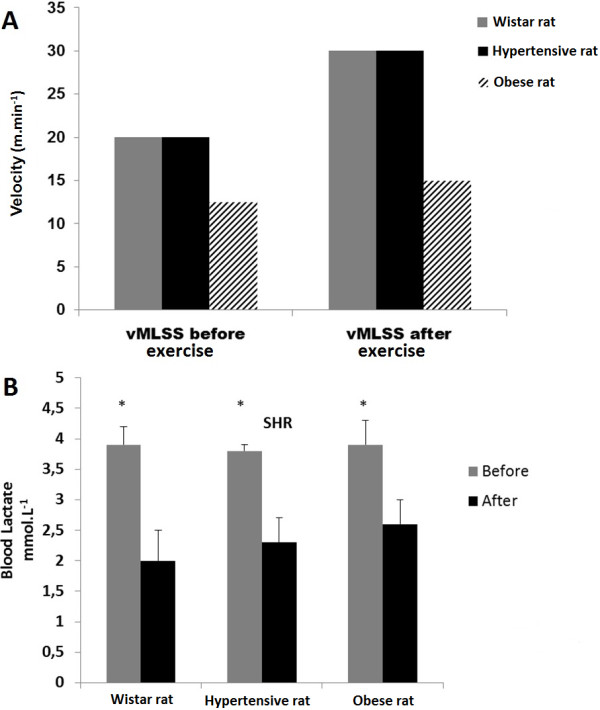
**Training parameters.** Comparison between exercise training velocity from MLSS (vMLSS) before and after four weeks of treadmill running exercise at moderate intensity, indicating that all animals from each group enhanced their aerobic capacity as demonstrated by improvement in the MLSS corresponding to velocity (15 m.min^−1^ for Obese rats and 3 0 m.min^−1^ for Hypertensive and Wistar rats) **(A)**. When the initial and final velocity of exercise training was compared, a significant reduction was evidenced in BLC of ~49% for Wistar rats, ~39% for Hypertensive rats and ~33% for Obese rats (p < 0.01) **(B)**.

After four weeks of treadmill running exercise at moderate intensity, a novel MLSS assessment evidenced that all animals from each group had enhanced their aerobic capacity as demonstrated by improvement in the MLSS corresponding velocity (15 m.min^−1^ for Obese rats and 30 m.min^−1^ for Hypertensive and Wistar rats) (Figure 1A). Furthermore, when comparing the initial and final velocity of exercise, a significant reduction was evidenced in blood lactate concentration (BLC) of ~49% for Wistar rats ~39% for Hypertensive rats and ~33% for Obese rats. This reduction in BLC (before vs. after exercise training) demonstrates the effectiveness of the proposed exercise training intensity for all animals (p < 0.01) (Figure 1B).

### Composition of fecal microbiota in rat lineages

After quality filtering, 889,124 out of 1,398,681 sequences were obtained from fecal samples collected pre-exercise and post-exercise, after 4 weeks of moderate exercise training (Additional file 1). An average of 49,951 denoised sequences per animal were obtained (average read length of 524.8) which composed an average of 583.1 distinct observed OTUs. Post-training samples presented a higher Shannon index compared to pre-training samples (6.4 ± 0.5 vs. 6.8 ± 0.2) (Additional file 3). Detailed sequencing information from all individual rats is presented in (Additional file 3).

Bacterial diversity was assessed by rarefaction measure of observed species against the number of sequences per sample, and the observed OTUs were identified with 97% of identity. Here, the rarefaction measure showed that species diversity (Additional file 4: A; Wistar rats; B; Hypertensive rats and C; Obese rats) reached a plateau tendency in all samples as the number of sequences increased, indicating that in the present study more sequences are unlikely to yield many additional species. As demonstrated in Additional file 4, the rarefaction curves revealed that OTU richness in post-exercise fecal samples is more species-rich than those found in the pre-exercise fecal samples. This was further evidenced in hypertensive rats and obese rats samples (Additional file 4B and C).

The relative abundance of the main dominant bacterial phylum from all fecal samples collected before and after exercise training is shown in Additional file 5A. Here, Firmicutes and Bacteroidetes are the most dominant phyla, followed by Proteobacteria. Firmicutes was shown to be enhanced after exercise training (1.1 fold change, p < 0.05) (Additional file 5B), thus being more evidenced in Obese rats (Obese rats; 0.69 ± 0.03 vs. Exercised Obese rats; 0.78 ± 0.04, p < 0.05). On the other hand, Proteobacteria were shown to be 1.8 fold reduced after exercise training (p < 0.05) (Additional file 5C). The Bacteroidetes phylum was shown to be 1.3 fold reduced after exercise only in Wistar rats (Wistar rats; 0.23 ± 0.04 vs. Exercised Wistar rats; 0.17 ± 0.03, p < 0.05).

### Composition of bacterial communities before, during and after exercise training

**Figure 2:**
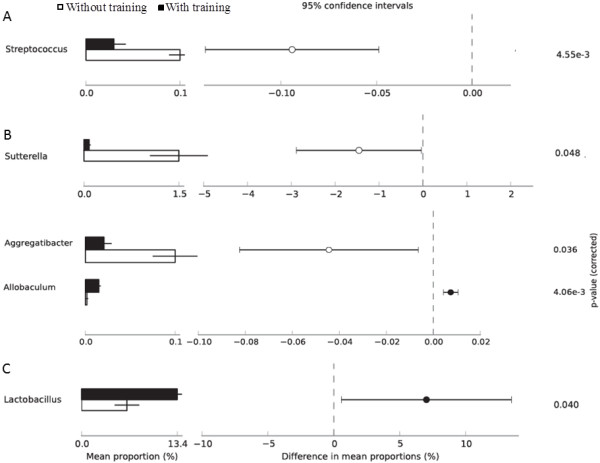
**Effect of exercise on Genus relative abundance.** Proportion of relative abundance for the statistical analyses of Genus level profiles distributed in Wistar rats **(A)**, Hypertensive rats **(B)** and Obese rats **(C)**. The genera were altered between without exercise training (white bar) and with exercise training (black bar). Features with a *α-*value of < 0.05 were considered significant.

The relative abundance at bacterial genus level for all animal lineages in response to exercise training was assigned only to those that presented a minimum variation at significant level (p < 0.05) (Figure 2). In Wistar rats (A), Streptococcus was the only genus that presented a significant alteration within its abundance, while untrained rats were more enriched with Streptococcus when compared to post-exercise (p < 0.05) (Figure 2A). In hypertensive rats, three genera (Allobaculum, Aggregatiobacter and Sutterella) were shown to be altered by exercise training. Despite minimal variation in the relative abundance of Allobaculum between pre-exercise and post-exercise samples, this genus was enriched by exercise training (p < 0.05) (Figure 2B). This was in contrast to Allobaculum, Aggregatibacter and Suturella where both were more abundant in pre-exercise samples (Figure 2B). Aggregatibacter presented a minimal variation in their relative abundance in fecal samples pre- and post-exercise training; however, exercise was shown to reduce the abundance of this genus (p < 0.05) (Figure 2B). The Suturella genus was also shown to be more enriched pre-exercise, with a greater relative proportion of all in this genus in hypertensive rats (p < 0.05). In Obese rats, Pseudomonas and Lactobacillus were both significantly altered after exercise training (Figure 2C). Minimal variation in Pseudomonas relative abundance was observed between samples (P < 0.05), while Lactobacillus presented the higher relative abundance after exercise from all identified genera (p < 0.05) (Figure 2C).

**Figure 3:**
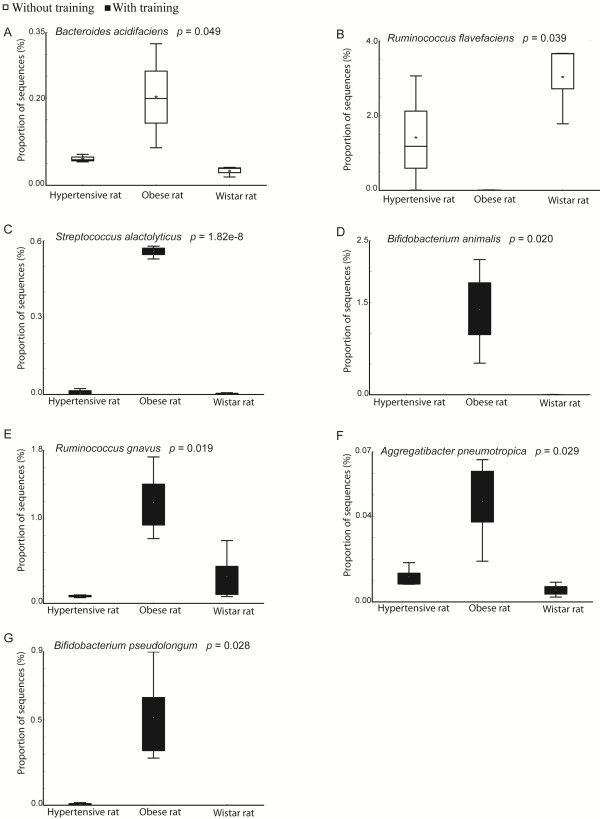
**Species abundance profile of fecal sample before and after exercise training.** Box plot showing the distribution in the proportion of sequences (%) of main species of each rat lineage (**A**; *Bacteroides acidifaciens*, **B**; *Ruminococcus flavefaciens*, **C** *; Streptococcus alactolyticus*, **D**; *Bifidobacterium animalis*, **E**; *Ruminococcus gnavus*, **F**; *Aggregatibacter pneumotropica*, **G**; *Bifidobacterium pseudolongum*) without exercise training (white box) and with exercise training (black box). The median value is shown as a line within the box and the mean value as a star, p-value for statistical significance was defined as p ≤ 0.05.

The proportion of sequences (%) of the main bacterial species from fecal samples collected before and after exercise training is shown in box plots (Figure 3). In pre-exercise fecal samples (Figure 3A and B), only two species (*Bacteroides acidifaciens* and *Ruminococcus flavefaciens*) presented a significant differential abundance, in contrast to fecal samples post-exercise training, where six species (*Streptococcus alactolyticus, Bifidobacterium animalis, Ruminococcus gnavus, Aggregatibacter pnemotropica* and *Bifidobacterium pseudolongum*) presented a differential abundance (Figure 3C-G). From all samples (pre and post-exercise) only one species (*Ruminococcus flavefaciens*) was less abundant in obese animals (Figure 3B), with all other species being significantly more enriched in the obese animals (Figure 3C-G).

Regarding pre-exercise samples, the proportion of sequences from the *Bacteroides acidifaciens* species was significantly more abundant in Obese rats than in Wistar and Hypertensive rats (p < 0.05) (Figure 3A). However, an opposite profile is observed in sequences attributed to the *Ruminococcus flavefaciens* species, where greater abundance is seen in Wistar rats followed by Hypertensive rats, with no abundance seen in Obese rats (p < 0.05) (Figure 3B). After exercise training, the proportion of sequences indicated that all listed species (*Streptococcus alactolyticus, Bifidobacterium animalis, Ruminococcus gnavus, Aggregatibacter pneumotropica* and *Bifidobacterium pseudolongum*) were more abundant in Obese rats compared to Wistar and Hypertensive rats lineages (Figure 3C-G respectively). The relative abundance of *Streptococcus alactolyticus* in Obese rats diverged significantly from Hypertensive and Wistar rats (p < 0.05), while a diminished proportion of sequences was seen in both rat strains (Figure 3C). The *Bifidobacterium animalis* species was seen to be highly enriched in Obese rats (p < 0.05) and absent in Wistar and Hypertensive rats (Figure 3D). In relation to R*uminococcus gnavus,* this species was poor in Wistar and almost absent in Hypertensive rats, but more abundant in Obese rats **(**Figure 3E). The *Aggregatibacter pneumotropica* species presented a similar profile to the previous species, being also more abundant in Obese rats compared to Wistar and Hypertensive rats (p < 0.05) (Figure 3F). Lastly, from the Actinobacteria phylum, *Bifidobacterium pseudolongum* abundance was almost exclusive to Obese rats (p < 0.05), being almost absent in Hypertensive rats and completely absent in Wistar rats group (Figure 3G).

### Principal coordinates analysis (PCoA)

**Figure 4:**
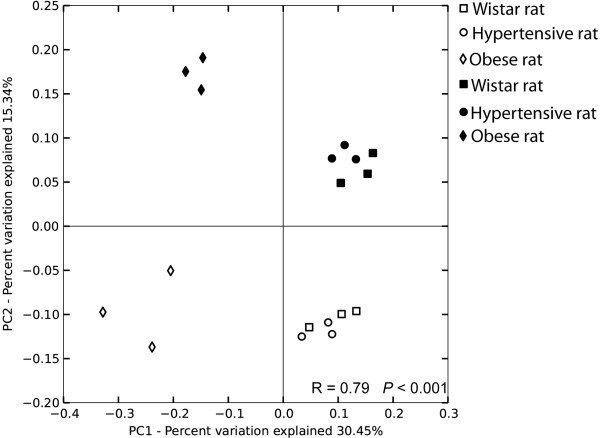
**Effect of exercise training on bacterial community.** Principal coordinates analysis (PCoA) of unweighted UniFrac distances generated from fecal samples in Wistar rats (squares), Hypertensive rats (circles) and Obese rats (diamonds) collected from triplicate rats without exercise training (white symbols) and with exercise training (black symbols). The result of the ANOSIM similarity analyses confirmed that samples harbor a distinct bacterial community.

Principal coordinates analysis (PCoA) of unweighed UniFrac distances was calculated and compared between all fecal samples collected pre and post-exercise from the three rat lineages in order to observe similarity in microbiota composition and the effect of exercise training (Figure 4). All three biological replicates from each animal lineage (Wistar, Hypertensive and Obese rats) were shown to cluster with a high correlation between them (R = 0.79, P < 0.001). UniFrac (PCoA) analysis showed that Wistar and Hypertensive rats share a similar bacterial composition, thus clustering far from Obese rats, indicating a distinct bacterial community composition between these rat lineages (Figure 4). It also indicated that microbiota from Wistar, Hypertensive and Obese rats were significantly altered by exercise training, where pre-exercise samples clustered significantly far from fecal samples collected after four weeks of exercise training (Figure 4). However, even though exercise altered microbial community composition in every animal lineage, Wistar and Hypertensive rats still maintained a similar bacterial composition, still clustering far from Obese rats microbiota (Figure 4).

### Correlation of bacterial abundance and lactate concentration

**Figure 5:**
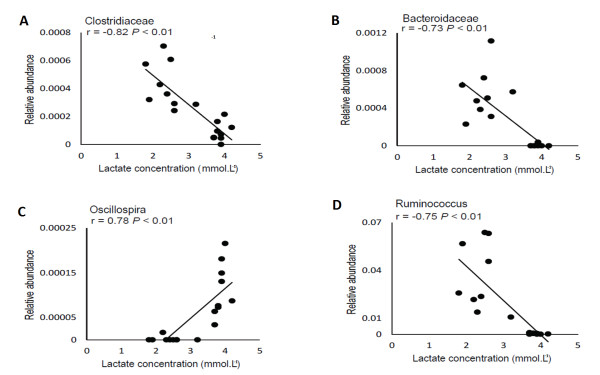
**Microbial abundance and blood lactate concentration correlation.** Correlations between the relative abundances of the bacterial communities (OTUs) and blood lactate concentration (mmol.L^−1^). Gut microbiota profile was determined by 16S rRNA pyrosequencing for Wistar rats, Hypertensive rats and Obese rats in fecal samples. Pearson correlation coefficients (*r*) are shown for each taxon (**A**; Clostridiaceae, **B**; Bacteroidaceae, **C**; Oscillospira, **D**; Ruminococcus), with the associated FDR-corrected *P* values.

As shown in Figure 1A, when comparing the initial and final velocity of exercise training, a significant reduction in BLC was evidenced in all rat lineages, where lower BLC (means of 2.3 mmol.L^−1^, Figure 1B) is associated to an improved aerobic capacity from trained status when compared to higher BLC (3.8 mmol.L^−1^) from untrained rats (pre-exercise samples from all rat lineages). Therefore, fecal bacterial communities were plotted against BLC to establish a correlation between microbial abundance and training status (Figure 5). OTUs from two bacterial families (Clostridiaceae and Bacteroidaeae) and two genera (Oscillospira and Ruminococcus) were found to be significantly correlated with BLC. The OTU abundance from both bacterial families was negatively correlated to BLC (Clostridiaceae, R = −0.82 P < 0.01; Bacteroidaceae, R = −0.73 P < 0.01). In both cases, higher abundance in OTUs was observed to correlate with lower lactate concentrations, indicating that exercise training may be favorable to the proliferation of these OTUs from both bacterial families (Figure 5A and B). The relative abundance of OTUs from Bacteroidaceae family was shown to be close to zero when BLC reached ~4 mmol.L^−1^ (Figure 5B), being associated with untrained status. Regarding the genera, the abundance of OTUs from Oscillospira and Ruminococcus genera presented divergent correlations with BLC. OTUs from Oscillospira were shown to be positively correlated to BLC (R = 0.78 P < 0.01) and Ruminococcus was negatively correlated (R = −0.75 P < 0.01) (Figure 5C and D). The OTUs from Oscillospira genus were shown to be almost absent in lower lactate concentrations, increasing their abundance with higher concentrations over 3.5 mmol.L^−1^, which indicates that exercise training may be seen as an unfavorable factor for a specific OTU from Oscillospira genus and its proliferation in gut environment (Figure 5C). However, the OTUs from Ruminococcus microbial genus were shown to be more abundant at lower lactate concentration and with almost no abundance at higher BLC, indicating that exercise training may also influence proliferation in this genus.

## Discussion

Several environmental [44] and host-related factors [16] are known to affect gut microbiota composition. This dynamic ecosystem, highly susceptible to external agents, has a symbiotic link with host health homeostasis. In this context, imbalanced gut microbiota has been associated with the development of inflammatory gastrointestinal diseases, obesity and altered metabolic status [19].

Recently, physical activity was shown to modulate gut microbiota in diet induced obesity [24], and in healthy rodents, altering the microbiota diversity and composition [23] and increasing n-Butyrate concentration in the cecum [22]. In contrast to some of these previous studies that used PCR-TGGE [22] and PCR-DGGE of bacterial 16S rRNA genes [23], here a robust pyrosequencing of the 16S rRNA genes was used, along with controlled exercise training parameters to investigate this relationship in non-pathologic and pathologic rat models.

Rarefaction measurements and Shannon index indicated that exercise training enhances bacterial diversity in non-pathological Wistar rats and in Obese and Hypertensive rats (Additional file 3 and Additional file 4). Here, Firmicutes and Bacteroidetes were found to be the most predominant phyla in all animal lineages (Additional file 5A). This predominance was also seen in mice cecum [6], and in exercised rats [23]. Considering all rat lineages, exercise was shown to enhance Firmicutes abundance and to diminish Proteobacteria content (Additional file 5B, C). Thus, Firmicutes were more abundant in post-exercise samples compared to pre-exercise in obese rats (Obese rats; 0.69 ± 0.03 vs. Exercised Obese rats; 0.78 ± 0.04, p < 0.05), while Bacteroidetes was shown to be reduced after training only in WR (Wistar rats; 0.23 ± 0.04 vs. Exercised Wistar rats; 0.17 ± 0.03, p < 0.05). Moreover, Bacteroidetes have been reported to be diminished in obese mice [10], while the ratio of Firmicutes to Bacteroidetes was shown to change in favor of Bacteroidetes in overweight and obese subjects, compared to the lean group [45]. Thus, as previously stated by Harris et al., [7] studying gut microbiota and their relation with metabolic disorders revealed that there is no difference between obese and lean individuals at phylum level. However, data here reported showed a significant alteration in bacterial community abundance at phylum as well as at genus levels (Figure 2), which could be associated with the effects of exercise and/or pathological conditions.Furthermore, our study revealed a significant alteration in bacterial community abundance at genus and species level as an effect of exercise and/or pathological stimuli (Figure 2).

In accordance with the PCoA analysis presented in this study (Figure 4), other studies have also reported a distinction between non-obese and obese microbiota from Zucker^fa/fa^ rats [46] and ob/ob mice [10]. We also reported that Wistar and Hypertensive rats share a similar microbiota composition (Figure 4). In a similar way, it was reported that rats treated with nitric oxide synthase inhibitor NG-nitro-L-arginine methyl ester (L-NAME) develop hypertension, with a variation in cecal microbiota compared to control normotensive rats [18]. Regarding the effect of exercise, PCoA analysis demonstrated that four weeks of moderate exercise training significantly altered microbiota composition in all rat lineages (Figure 4). According to our results, different exercise volumes, 6 days [23], and 5 [22] and 12 weeks [24] of voluntary running exercise, were shown to alter microbiota composition, indicating that the microbial community is affected even by a few days of exercise. Together, these data suggest that besides other well-known factors, exercise may be seen as a potential environmental factor capable of modulating gut microbiota.

Here, exercise was also shown to significantly alter six bacterial genera (Figure 2). Fecal samples were more enriched with Allobaculum (Hypertensive rats), Pseudomonas (Obese rats) and Lactobacillus (Obese rats) after exercise training, while Streptococcus (Wistar rats), Aggregatibacter (Hypertensive rats) and Sutturela (Hypertensive rats) were shown to be more abundant before exercise training was performed **(**Figure 2). The Lactobacillus genus presented higher abundance after exercise only in Obese rats (13.4% p < 0.05) (Figure 2C). In agreement with our data, the recent study of Queipo-Ortuno et al. [23] revealed that Lactobacillus was also enhanced with exercise in lean rats, with a longer exercise stimulus (6 weeks). Lactic acid bacteria (LAB), represented in our study by Lactobacillus (enriched after exercise), are associated with the mucosal surface of the small intestine and colon in animals, where they produce lactic acid though homo or heterofermentative metabolism [47]. In this second process, besides lactic acid, CO_2_, acetic acid and/or ethanol are produced [48], which may contribute to a more acidic environment [48]. It has been reported that LAB in the gastrointestinal tract leads to positive health benefits with influence on microflora, modulation of mucosal immunity and exclusion of pathogens [47]. The enrichment of Lactobacillus in Obese rats after exercise may have some influence on gastrointestinal acidity trough the production of acidic compounds (e.g. lactic acid, acetic acid); however, this parameter was not measured in the present study. The capillary blood lactic acid was measured in order to establish aerobic capacity and thus to be used as a parameter for adaptation to exercise. Moreover, the lactate produced by Lactobacillus bacteria is converted into butyrate in the gut through bacteria such as *B. Coccoides and E. rectale,* also found to be enhanced after exercise [23]. Furthermore, butyrate is shown to be related to mucin synthesis and gut epithelium protection [49]. In another study, Matsunomo et al. [22] showed that exercise altered microbiota and enhanced n-butyrate concentration in rats’ cecum. Therefore, enhanced Lactobacillus found in Obese rats group may possibly have a positive role in the gastrointestinal environment of these animals. It has been reported that obesity-associated gut microbiota is enriched in some species of Lactobacillus (e.g. *Lactobacillus reuteri*) [50] while other species (e.g. *Lactobacillus gasseri* BNR17) are involved in metabolism regulation [51], presenting anti-obesity effects [52].

In our study, the Sutterela genus was more abundant before exercise training in Hypertensive rats (Figure 2B). The role of this genus in inflammatory bowel disease has been recently investigated with no relation being found [53]. Nevertheless, more research is needed to understand the relation of Sutturela with exercise and its possible gastrointestinal protective effect.

It is observed that these alterations in genera are not consistent across all host phenotypes. We believe that part of this inconsistency may be related to the biologic differences peculiar to each host genotype used in the study. Obesity has been shown to modulate gut microbiota [10, 11], while no study has shown this yet in a hypertensive phenotype. Furthermore, as a first exploratory study to use different genotypes with different pathologies, it is interesting to note that the gut microbiota of 3 different phenotypes (and 2 genotypes) was possibly altered by an external factor such as exercise.

Seven bacterial species were shown to have a significant differential abundance altered between the three animal lineages (Figure 3). From fecal samples collected pre-exercise, only *Bacteroides acidifaciens* and *Ruminococcus flavefaciens* presented a significant differential abundance (Figure 3A and B). While *Bacteroides acidifaciens* was more enriched in Obese rats compared to Wistar and Hypertensive rats (Figure 3A), *Ruminococcus flavefaciens* showed an opposite profile, being more enriched in Wistar rats followed by Hypertensive rats and significantly depleted in Obese rats (Figure 3B). *B acidifaciens* has recently been shown to have an important role in the production of imunoglobulin (IgA) in the large intestine of mice [54]. This production plays an adaptive role in the intestinal mucosal immune system [55]. Since IgA is enhanced in metabolic disorders [56], the relative abundance of *Bacteroides acidifaciens* in Obese rats (Figure 3A) may be associated with the role of gut microbiota in the inflammatory signalling peculiar to obesity [57]. Otherwise, *Ruminococcus flavefaciens* is a cellulolytic bacterium present in the rumen of mammals, and it has been shown to be inhibited by probiotic supplementation (*L. acidophilus* NCFM) in young rats [58]. Our data indicate that the obesity phenotype from obese rats may suppress this particular species.

In samples collected after training, *Streptococcus alactolyticus, Bifidobacterium animalis, Ruminococcus gnavus, Aggregatibacter pnemotropica* and *Bifidobacterium pseudolongum* were all more abundant in Obese rats (Figure 3C-G). *Streptococcus alactolyticus* and *Bifidobacterium animalis* species were shown to be present in the gut of obese rats. In contrast to our data, Bifidobacterium is often associated with lean phenotypes [19]; however, our study showed that this species was completely absent in non-obese Wistar rats and Hypertensive rats (Figure 3D). Regarding the relative abundance of *Ruminococcus gnavus* in Obese rats*,* this species is known to have an antibacterial effect and to protect the host from pathogens [59], also found to be reduced in colon cancer tissue [60]. However, this species was also shown to be enhanced in diverticulitis [61], which is commonly associated with obesity [62]. *Bifidobacterium pseudolongum* was another species almost exclusively in Obese rats (Figure 3G). Moreover, the content of this species was shown to be enhanced in obese mice induced by diet and probiotic administration, when compared to a group of mice without probiotic supplementation [63].

In the present study, the MLSS was used to assess aerobic improvement as a result of four weeks of exercise training at moderate intensity [2]. Thus, after exercise training, a significant reduction in BLC was observed in all rat lineages (Figure 1B), which is associated with an improved aerobic capacity when compared to higher BLC from untrained rats (Figure 1B). The OTUs from Oscillospira and Ruminococcus families were found to be negatively correlated with BLC (Clostridiaceae, R = −0.82, P < 0.01; Bacteroidaceae, R = −0.73 P < 0.01) as were the OTUs from Ruminococcus genus (R = −0.75 P < 0.01). In these three cases, the greatest relative abundance of OTUs in both families is correlated with lower BLC, indicating that exercise training may be favorable to the proliferation of these specific OTUs (Figure 5A, B and D). Otherwise, OTUs from Oscillospira presented a positive correlation with BLC (R = 0.78, P < 0.01) (Figure 5C). The relative abundance within these OTUs is seen to increase when the concentration of lactate goes over ~3.5 mmol.L^−1^. Since lower BLC during the MLSS test was associated with a more trained status, this result may indicate that exercise training may affect the abundance of the OTUs from this genus.

## Conclusions

These findings suggest that exercise training is capable of altering gut microbiota at genus level, with significant alteration in bacterial composition and diversity in obese, non-obese and hypertensive rats. Exercise was shown to enhance the relative abundance of three genera, with Lactobacillus being the most abundant, while another three genera were shown to be more abundant before exercise training (Streptococcus, Aggregatibacter and Sutterella). Non-obese Wistar rats and spontaneously hypertensive rats were shown to share similar microbiota, unlike Obese rats. Rat lineages were also shown to harbor a differential abundance at species level, and six species were shown to be significantly more abundant in obese rats. Two bacterial families (Clostridiaceae and Bacteroidaceae) and two genera (Oscillospira and Ruminococcus) were also shown to significantly correlate with blood lactate accumulation, while exercise was shown to be favorable to the two families and Ruminococcus genus in opposition to Oscillospira. In conclusion, this is the first study to use controlled exercise parameters to assess gut bacterial community modification in three different animal lineages, which may reflect the potential of exercise to alter gut microbial community. However, the effect of exercise on the acidity of lumen or fecal samples was not measured, which limits us in establishing a direct link between exercise and gut alteration by acidic induction. Thus, more studies are necessary to establish these modifications as possible therapeutic implications for obesity or hypertension treatment through the modulation of gut microbiota.

## Electronic supplementary material

Additional file 1: **Experimental design.** Obese rats (n = 3), Wistar rats (n = 3) and Hypertensive rats (n = 3) were used to verify the effect of four weeks of moderate exercise training on gut microbiota. Fecal samples were collected before and after exercise training. After DNA extraction, barcoded pyrosequencing of the rRNA genes was used to determine the gut microbiota modifications. (TIFF 909 KB)

Additional file 2: **Characterization of the hypertensive phenotype.** Histogram of blood pressure profile from Wistar rats and spontaneously Hypertensive rats measured by the tail-cuff method at the beginning of the experiment. Hypertensive rats showed a significantly higher systolic blood pressure (171.4 ± 7.7 mmHg) when compared to Wistar rats (128 ± 5.9 mmHg), indicating the hypertensive phenotype of this rat group. (TIFF 65 KB)

Additional file 3: **Table with the frequency of bacterial communities pre and post exercise training revealed by 16S rRNA pyrosequencing analysis.** (DOCX 15 KB)

Additional file 4: **Microbial alpha diversity.** Rarefaction curves for fecal samples, each with at least 23,000 16S rRNA sequences. Each line connects an average number (±SD) of observed 97% OTUs for **(A)** Wistar rats; **(B)** Hypertensive rats and **(C)** Obese rats. The color blue indicates the richness of bacterial communities without training and red indicates with training. (TIFF 150 KB)

Additional file 5: **Composition of fecal microbiota population of Wistar, Hypertensive and Obese rats before and after exercise training.** Bacterial distribution evaluated at the main phylum taxonomical level in fecal samples from Wistar, Hypertensive and Obese rats, collected from triplicate rats before and after four weeks of exercise training **(A)**. Results are shown for the independent samples described in Additional file 3, where the letter “E” represents the samples with exercise training. Boxplots of Firmicutes **(B)** and Proteobacteria **(C)** abundance pre and post-exercise. (TIFF 225 KB)
