# Supplementary figures and images for: Exercise induction of gut microbiota modifications in obese, non-obese and hypertensive rats
Source: BMC Genomics. 2014 Jun 21;15(1):511. doi: 10.1186/1471-2164-15-511 (PMC4082611; doi:10.1186/1471-2164-15-511)

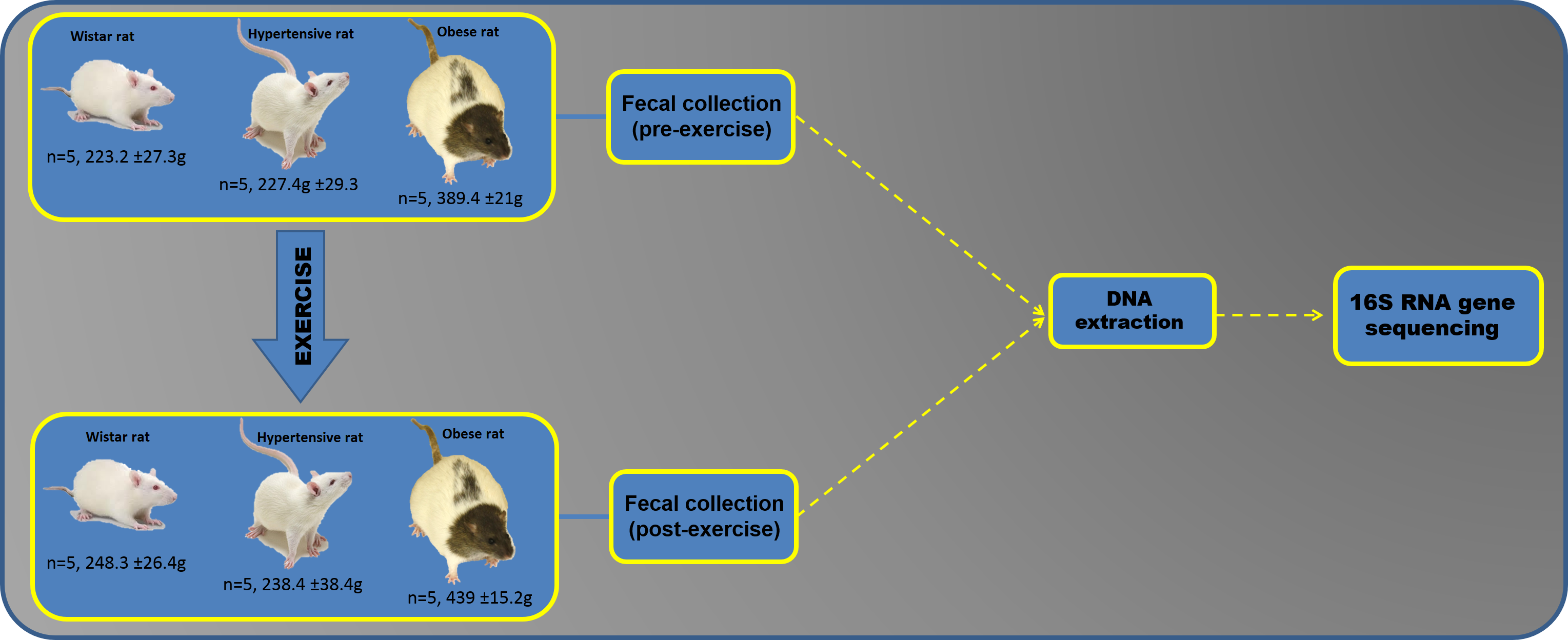

Supplement: Supplementary file 1 — Additional file 1: Experimental design. Obese rats (n = 3), Wistar rats (n = 3) and Hypertensive rats (n = 3) were used to verify the effect of four weeks of moderate exercise training on gut microbiota. Fecal samples were collected before and after exercise training. After DNA extraction, barcoded pyrosequencing of the rRNA genes was used to determine the gut microbiota modifications. (TIFF 909 KB) [file 12864_2013_6191_MOESM1_ESM.tiff]

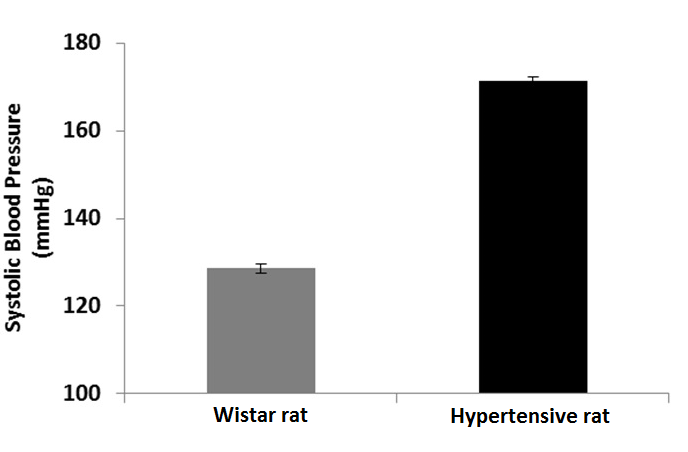

Supplement: Supplementary file 2 — Additional file 2: Characterization of the hypertensive phenotype. Histogram of blood pressure profile from Wistar rats and spontaneously Hypertensive rats measured by the tail-cuff method at the beginning of the experiment. Hypertensive rats showed a significantly higher systolic blood pressure (171.4 ± 7.7 mmHg) when compared to Wistar rats (128 ± 5.9 mmHg), indicating the hypertensive phenotype of this rat group. (TIFF 65 KB) [file 12864_2013_6191_MOESM2_ESM.tiff]

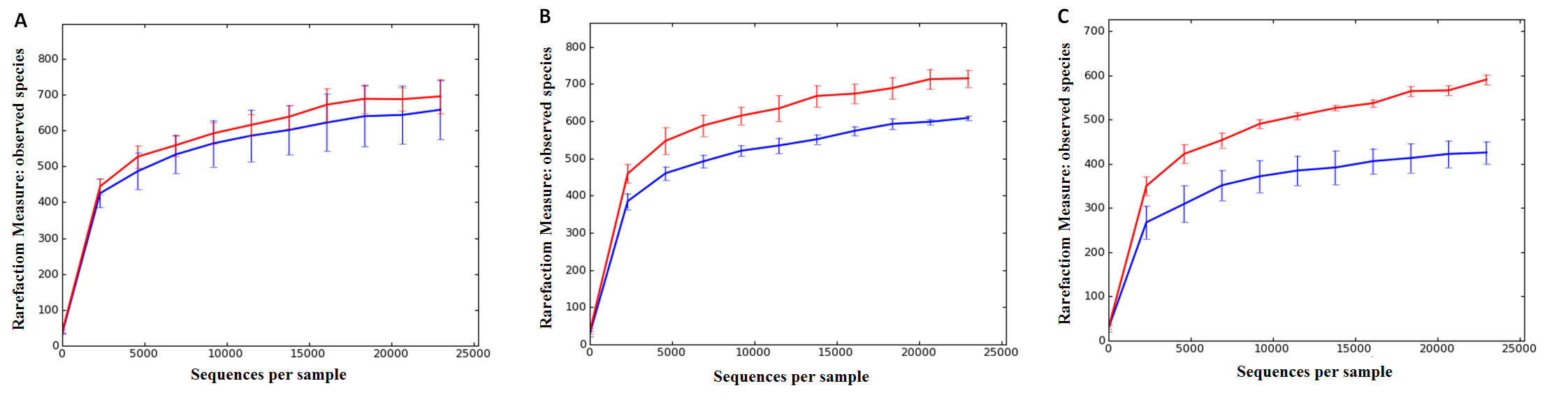

Supplement: Supplementary file 4 — Additional file 4: Microbial alpha diversity. Rarefaction curves for fecal samples, each with at least 23,000 16S rRNA sequences. Each line connects an average number (±SD) of observed 97% OTUs for (A) Wistar rats; (B) Hypertensive rats and (C) Obese rats. The color blue indicates the richness of bacterial communities without training and red indicates with training. (TIFF 150 KB) [file 12864_2013_6191_MOESM4_ESM.tiff]

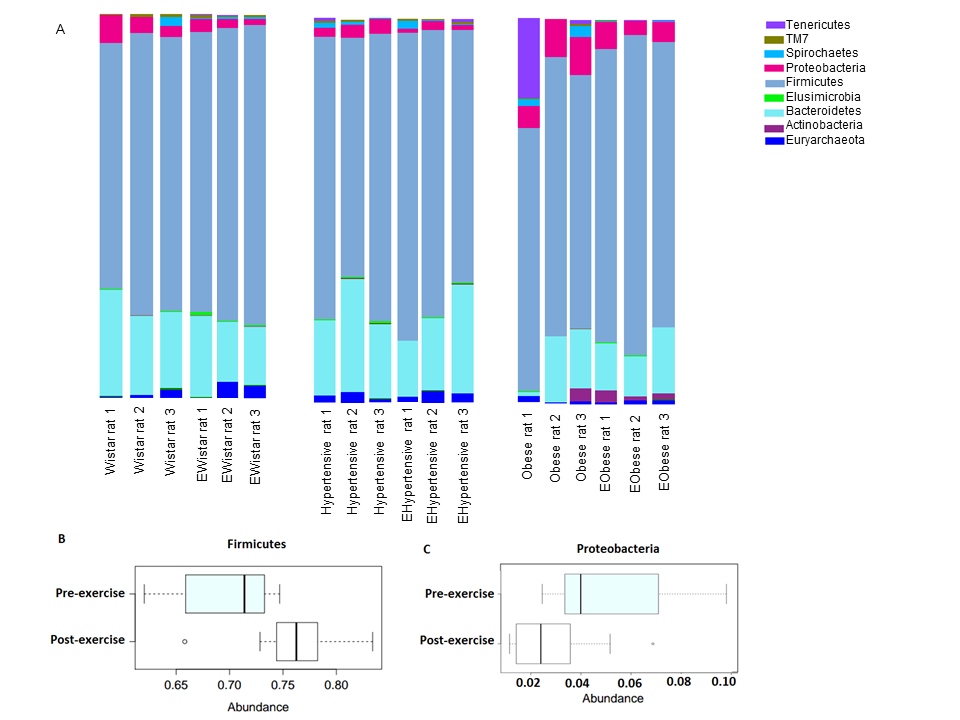

Supplement: Supplementary file 5 — Additional file 5: Composition of fecal microbiota population of Wistar, Hypertensive and Obese rats before and after exercise training. Bacterial distribution evaluated at the main phylum taxonomical level in fecal samples from Wistar, Hypertensive and Obese rats, collected from triplicate rats before and after four weeks of exercise training (A). Results are shown for the independent samples described in Additional file 3, where the letter “E” represents the samples with exercise training. Boxplots of Firmicutes (B) and Proteobacteria (C) abundance pre and post-exercise. (TIFF 225 KB) [file 12864_2013_6191_MOESM5_ESM.tiff]
