# Supplementary material for: Exercise induction of gut microbiota modifications in obese, non-obese and hypertensive rats
Source: BMC Genomics. 2014 Jun 21;15(1):511. doi: 10.1186/1471-2164-15-511 (PMC4082611; doi:10.1186/1471-2164-15-511)
Supplement: Supplementary file 3 — Additional file 3: Table with the frequency of bacterial communities pre and post exercise training revealed by 16S rRNA pyrosequencing analysis. (DOCX 15 KB) [file 12864_2013_6191_MOESM3_ESM.docx]

| **Sample ID** | **Treatment** | **No. of initial seqs** | **No. of final seqs** | **OTU_observed_(0.03)_** | **Shannon index** | **Avg. Read Length** |
| --- | --- | --- | --- | --- | --- | --- |
| Wistar rat1 | Pre-training | 41.185 | 26.760 | 501 | 6.26 | 526 |
| Wistar rat 2 | Pre-training | 41.911 | 23.184 | 549 | 7.08 | 529 |
| Wistar rat 3 | Pre-training | 97.296 | 57.362 | 756 | 7.03 | 526 |
| Hypertensive rat 1 | Pre-training | 96.930 | 63.069 | 631 | 6.82 | 525 |
| Hypertensive rat 2 | Pre-training | 86.732 | 54.951 | 605 | 6.37 | 524 |
| Hypertensive rat 3 | Pre-training | 98.934 | 68.876 | 619 | 6.03 | 524 |
| Obese rat 1 | Pre-training | 111.659 | 59.302 | 410 | 5.43 | 523 |
| Obese rat 2 | Pre-training | 64.952 | 52.432 | 391 | 5.79 | 523 |
| Obese rat 3 | Pre-training | 64.977 | 44.150 | 454 | 6.45 | 524 |
| E Wistar rat1 | After training | 72.145 | 43.398 | 624 | 6.95 | 527 |
| E Wistar rat2 | After training | 92.674 | 59.077 | 680 | 6.94 | 529 |
| E Wistar rat3 | After training | 72.799 | 44.001 | 715 | 6.70 | 526 |
| E Hypertensive rat 1 | After training | 90.997 | 60.669 | 693 | 6.91 | 527 |
| E Hypertensive rat 2 | After training | 71.630 | 43.615 | 723 | 7.12 | 525 |
| E Hypertensive rat 3 | After training | 71.600 | 47.042 | 680 | 6.57 | 524 |
| E Obese rat 1 | After training | 82.287 | 57.836 | 562 | 6.56 | 524 |
| E Obese rat 2 | After training | 84.030 | 55.752 | 574 | 6.63 | 521 |
| E Obese rat 3 | After training | 55.943 | 37.648 | 509 | 6.43 | 521 |
